# Organizational Silence and Related Factors Among Shift Work Nurses in Korea: A Cross-Sectional Study

**DOI:** 10.1155/jonm/1256556

**Published:** 2025-11-19

**Authors:** Sung Eon Sim, Hye-Young Jang

**Affiliations:** Department of Nursing, College of Nursing, Hanyang University, Seoul, Republic of Korea

**Keywords:** bullying, communication, interpersonal relations, nurses, personnel management, professional autonomy, self-concept, workplace

## Abstract

**Aim:**

This study aimed to explore factors contributing to organizational silence, drawing upon the framework of the choice to remain silent by Milliken, Morrison, and Hewlin.

**Background:**

The promotion of open communication and the enabling of open discourse amongst nursing professionals are essential for the enhancement of health service quality and the assurance of patient safety.

**Methods:**

A cross-sectional descriptive survey was conducted from August 14, 2023, to September 15, 2023. Data were collected from 170 rotating shift nurses at a tertiary hospital in South Korea. The hypotheses were tested using multiple regression and the PROCESS macro, Version 4.2, Model 4.

**Results:**

Leader–member exchange not only had a direct effect on organizational silence but also had a significant indirect effect mediated by workplace bullying (*β* = −0.03, 95% CI: [−0.074, −0.001]). The total effect was confirmed to be *β* = −0.23 (95% CI: [−0.345, −0.129]).

**Conclusion:**

Organizational silence is shaped by both individual characteristics and organizational conditions. Workplace bullying not only has a direct effect on organizational silence but also mediates the relationship between leader–member exchange and organizational silence.

**Implications for Nursing Management:**

Nurse managers should be sensitive to negative cultures such as bullying and actively promote leader–member and team-member exchange. When nurse managers actively maintain and support relationships with members, members are more likely to disclose issues and express their opinions.

## 1. Introduction

The successful implementation of organizational change is contingent upon the voices of its members. The challenge, however, is that although members are aware of issues within the organization or their superiors' decisions, they tend to remain silent rather than express their opinions on these matters [1]. Organizational silence is defined as the intentional withholding of ideas, opinions, and information that could contribute to improving the organization [2, 3]. In Korea, a deeply rooted hierarchical and authoritarian culture contributes to a perception that speaking up is risky or inappropriate [4, 5]. This norm is especially prevalent in nursing, where rigid seniority structures discourage open communication and reinforce silence [6]. Especially, given the nature of shift nurses, where patient information is shared through handoffs, silence can lead to serious problems. Shift nurses are particularly vulnerable to the negative effects of silence. Given the necessity of frequent handovers, omitting critical patient information due to silence can jeopardize care quality and patient safety [7, 8]. Understanding organizational silence in this group is thus of practical significance.

In Korean nursing settings, nurses often remain silent due to fear of retaliation or doubts about the efficacy of speaking up [6]. Workplace bullying, especially the culturally unique form known as Taeum, further reinforces silence by instilling fear and preserving hierarchy [9, 10]. Despite its importance, few studies have explored organizational silence within the cultural context of Korean nursing or applied theory-driven models to identify its determinants [4, 11].

This study applies Milliken et al.'s [1] model to examine individual and organizational factors influencing silence among Korean shift nurses. Focusing on professional autonomy, self-esteem, LMX, TMX, and workplace bullying, the study adopts an integrative approach by addressing both suppressors and triggers of silence. These modifiable variables offer practical insights for interventions aimed at improving communication, work environments, and patient safety.

## 2. Theory and Hypotheses

### 2.1. Organizational Silence

Organizational silence refers to behaviors when members consciously choose to withhold their opinions, either due to fear of negative consequences or to avoid discomfort or awkwardness [2]. Organizational silence is typically classified based on the underlying motivations, as outlined in Dyne et al.'s taxonomy [3, 5]. Members of an organization, motivated by various factors, may opt for silence because of a lack of belief that their input will result in organizational change, which is a phenomenon termed acquiescent silence. On the other hand, defensive silence occurs when individuals choose to remain silent to avoid potential negative repercussions from their own statements. Prosocial silence, conversely, occurs when individuals decide to stay silent to prevent potential harm to their colleagues or the organization [3].

**Figure 1:**
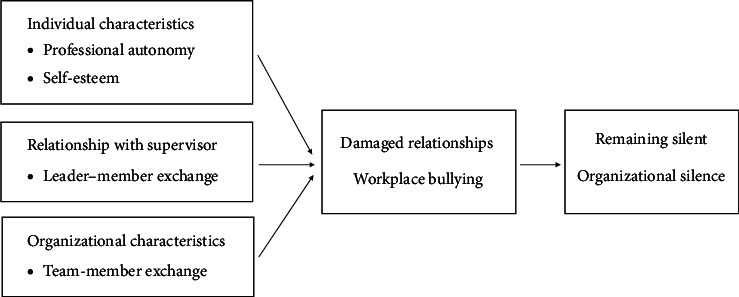
Diagram of this study.

Milliken et al. [1] proposed the “Model of the choice to remain silent,” suggesting that individuals opt to remain silent in organizations due to negative expectations arising from individual characteristics, relationships with supervisors, and organizational attributes. This model is instrumental in structuring relationships between research variables to comprehend the impact of organizational silence. Our study designed a framework based on this model (Figure 1).

### 2.2. Workplace Bullying as a Mediating Role

Milliken et al. [1] argued that the anticipation of negative consequences directly leads to silence and also mediates other contributing factors, emphasizing the psychological risks associated with speaking up. To examine this mechanism, the present study explores workplace bullying as a mediating variable between organizational factors and silence. Workplace bullying remains a widespread issue in nursing organizations worldwide [12]. In Korea, it has emerged as a serious social issue, contributing to nurses' suicides. Studies indicate that Korean nurses often refrain from speaking due to fear of negative social consequences, including physical abuse, social exclusion, and humiliation [13]. One qualitative study revealed, “Even if there is a justifiable reason, I choose to remain silent because I am afraid of being scolded for talking back” [9]. Such evidence highlights the strong connection between bullying and silence in nursing settings. Notably, research suggests bullying is most prevalent during handovers [10], placing shift nurses at particular risk. A noteworthy finding in prior research is that, according to the grounded theory study by Kang and Yun [14], the causal condition of workplace bullying was identified as poor job performance that falls short of expectations, while the contextual conditions included individual dispositions such as passivity and a hierarchical and rigid nursing organizational culture. This is consistent with the “Model of the choice to remain silent” proposed by Milliken et al. [1], which posits that individual characteristics, relationships with leaders, and organizational culture foster negative expectations that in turn mediate the emergence of silence. In this context, workplace bullying can be understood as a critical mediating mechanism through which personal and organizational factors jointly reinforce organizational silence. Prior studies have also found that bullying mediates the relationship between certain organizational conditions and silence [15]. Accordingly, this study posits workplace bullying as a key mediating mechanism in the selection pathway of organizational silence.

### 2.3. Organizational Silence and Individual Factors: Professional Autonomy and Self-Esteem

Milliken et al. [1] identified individual characteristics such as lower hierarchical status and lack of experience as key contributors to organizational silence. However, a Korean meta-analysis found that few studies incorporated such traits beyond basic demographics such as age or tenure [4, 11]. These variables provide limited insight into the psychological or dispositional factors that more directly influence silence. To address this gap, the present study focuses on professional autonomy and self-esteem as meaningful individual characteristics.

In this study, professional autonomy was chosen to explain nurses' lack of experience. In the medical profession, it is considered a personal trait that facilitates effective responsibility management [16]. Professional autonomy, particularly for nurses, is a core requirement worldwide, including adequate participation in decision-making processes and the right to formally challenge unfair practices [17]. Higher autonomy fosters confidence, encourages problem-solving, and may reduce silence. Indeed, greater job autonomy has been negatively linked to acquiescent and defensive silence in previous studies [18]. In Korean nursing settings, however, perceived incompetence is often met with reprimands or bullying [9, 10], which may discourage self-expression. Moreover, nurses may feel less competent or hesitant to express their opinions [6, 19]. Previous research has shown that job autonomy interacts with workplace bullying to significantly predict emotional exhaustion and depersonalization. Furthermore, workplace autonomy has been found to moderate the relationship between workplace bullying and organizational silence [20, 21]. This suggests that autonomy acts as a protective factor, mitigating the effects of negative organizational experiences on psychological distress and silence.

In addition, Morrison and Milliken [2] noted that there are not only cognitive but also emotional factors in the selection process for organizational silence. Pinder and Harlos [22] reported that individuals with low self-esteem and high communication anxiety exhibited silent behaviors more frequently. However, personality traits remain overlooked in organizational silence studies. Self-esteem reflects the degree to which one accepts oneself or has an emotional connection to self-worth and indicates how much one values oneself [23]. Although research on self-esteem's direct impact on organizational silence is limited, a previous study conducted in Türkiye [24] found that self-esteem significantly lowered both acquiescent silence and defensive silence. Moreover, individuals with high self-esteem are less likely to be targets of workplace bullying, which is a known antecedent of silence [25]. Thus, this study posits that workplace bullying may mediate the relationship between both professional autonomy and self-esteem with organizational silence. This study proposes the following hypotheses by applying professional autonomy and self-esteem as individual characteristics.

• H1: Workplace bullying mediates the relationship between professional autonomy and organizational silence.
• H2: Workplace bullying mediates the relationship between self-esteem and organizational silence.

### 2.4. Organizational Silence and Organizational Factors: Leader–Member Exchange (LMX) and Team-Member Exchange (TMX)

The silence of organizational members in certain situations means “not just silence,” but “a failure in upward communication” in terms of organizational development [2, 5, 6]. Milliken et al. [1] highlighted organizational factors such as supervisor relationships and a nonsupportive peer culture as the key contributors to organizational silence. In Korea, silence often stems from reluctance to challenge superiors or peers, with negative leader relationships being a key factor [5, 6]. Conversely, trust in leaders reduces the fear of speaking up and emotional uncertainty. Leaders play a crucial role in either fostering or inhibiting organizational silence [6, 11, 18, 26, 27]. In this regard, LMX plays a pivotal role in reducing workplace bullying and promoting psychological safety and promotes open communication [28, 29]. LMX, which emphasizes reciprocal interactions rather than hierarchical command, provides a valuable framework for understanding leadership's impact on silence [30].

Nurses work in shifts and provide 24-h care to patients. Owing to the nature of their work, they maintain interdependent relationships with their colleagues, share responsibilities, and exchange patient information and opinions about patients [31]. This demonstrates the unique significance of the “Team” concept in nursing organizations. TMX encompasses information sharing, mutual support, cooperation, and emotional trust among colleagues [32] and effectively represents the colleague relationships of nursing teams. High-quality TMX promotes open communication and reduces silence and has been associated with improved member attitudes and outcomes [33, 34]. While direct evidence linking TMX and workplace bullying is limited, studies suggest that bullying among nurses occurs not only in vertical relationships but also frequently in horizontal relationships among peers [35], and that strong team skills can help mitigate such behaviors [36]. Therefore, TMX is proposed as a key relational factor influencing both workplace bullying and organizational silence. Based on the previous literature review, we propose the following hypotheses:

• H3: Workplace bullying mediates the relationship between LMX and organizational silence.
• H4: Workplace bullying mediates the relationship between TMX and organizational silence.

## 3. Materials and Methods

### 3.1. Study Design and Participants

This descriptive study was conducted to identify factors influencing organizational silence and the mediating role of workplace bullying among nurses working rotating shifts at a tertiary hospital. The study was based on the model of the choice to remain silent by Milliken et al. [1] (Figure 1) and followed the Strengthening the Reporting of Observational Studies in Epidemiology (STROBE) guidelines (https://www.strobe-statement.org) [37].

Convenient sampling was used to recruit the nurses from H University Hospital in Seoul (855 beds and 926 nurses). Inclusion criteria included nurses working shifts, who had at least 6 months of clinical experience, and were directly providing patient care. Nurse managers and nonshift nurses were excluded. The required sample size was calculated using the G∗Power 3.1.9.7 program (Heinrich Heine University Düsseldorf, Düsseldorf, Germany) with a power of 0.80, a significance level of 0.05, and 13 predictors (sex, age, education, marital status, religion, department, clinical career, current work unit experience, professional autonomy, self-esteem, LMX, TMX, and workplace bullying). Based on the recommendation of a prior study [38], a 30% dropout rate was taken into account, leading to the recruitment of 190 participants. Of these, after excluding 20 responses due to incompleteness or insincerity, 170 valid questionnaires (89.5%) were included in the final analysis.

### 3.2. Measurements

#### 3.2.1. Organizational Silence

The organizational silence was measured using the instrument developed by Dyne et al. [3] and translated by Kang and Go [5]. This instrument consists of three subscales, encompassing a total of 15 items. These include five items each for acquiescent silence, where individuals withhold communication due to a lack of expectation for organizational change; defensive silence, where individuals avoid speaking for fear of negative consequences; and prosocial silence, where individuals choose not to speak for the benefit of colleagues or the organization. The responses were measured on a five-point Likert scale, from 1 (*strongly disagree*) to 5 (*strongly agree*), with higher scores indicating greater organizational silence. Milliken et al. [1] emphasized that the decision to remain silent is mainly made in a threat-avoidance context, such as fear of negative stigma, damage to relationships, and retaliation. The model of the choice to remain silent [2] explains acquiescent and defensive silence as forms of silence motivated by self-protection and fear-based concerns. In contrast, prosocial silence stems from positive and altruistic motivations that lie outside this framework. Moreover, the workplace bullying examined in this study reflects the hierarchical oppression and fear-based culture of Korean nursing organizations [39], which is closely tied to threat-avoidance motives for silence. Accordingly, prosocial silence was excluded from this study to maintain conceptual clarity and theoretical consistency. According to Kang and Go [5], the Cronbach's *α* was 0.87 for acquiescent silence and 0.92 for defensive silence. In this study, the Cronbach's *α* was 0.86 and McDonald's *ω* was 0.85.

#### 3.2.2. Workplace Bullying

The Workplace Bullying in Nursing-Type Inventory (WPBN-TI), developed by Lee and Lee [40], was used in this study. This instrument comprises 16 items rated on a 5-point Likert scale, from 1 (*strongly disagree*) to 5 (*strongly agree*), with higher scores indicating greater perceived workplace bullying. In the study by Lee and Lee [40], Cronbach's *α* was 0.91, while in this study, Cronbach's *α* was 0.90 and McDonald's *ω* was 0.90.

#### 3.2.3. Professional Autonomy

The Professional Autonomy Scale (PAS), developed by Schutzenhofer [41] and translated by Kim [16], was used in this study. The PAS comprises 11 items rated on a 5-point Likert scale, from 1 (*strongly disagree*) to 5 (s*trongly agree*). Higher scores indicate greater professional autonomy. Kim's study [16] reported a Cronbach's *α* of 0.80, while in this study, the Cronbach's *α* was 0.86 and McDonald's *ω* was 0.86.

#### 3.2.4. Self-Esteem

Self-esteem was measured using the Rosenberg Self-Esteem Scale (RSES), developed by Rosenberg [23] and translated by Jeon [42]. This instrument comprises 10 items rated on a 4-point Likert scale, from 1 (*strongly disagree*) to 4 (*strongly agree*), with higher scores indicating greater self-esteem. Cronbach's alpha was 0.85 in the study by Jeon [42], while in this study, the Cronbach's *α* was 0.88 and McDonald's *ω* was 0.87.

#### 3.2.5. LMX

LMX was measured using the instrument developed by Graen and Uhl-Bien [43] and translated by Yi and Yi [44]. This instrument comprises eight items rated on a five-point Likert scale, from 1 (*strongly disagree*) to 5 (*strongly agree*), with higher scores indicating greater LMX. Cronbach's alpha was 0.91 in the study by Yi and Yi [44], and in this study, the Cronbach's *α* was 0.93 and McDonald's *ω* was 0.93.

#### 3.2.6. TMX

TMX was measured using the instrument developed by Seers et al. [32] and modified by Im [45]. This instrument comprises ten items rated on a five-point Likert scale, from 1 (*strongly disagree*) to 5 (*strongly agree*), with higher scores indicating greater TMX. In Im's study [45], Cronbach's alpha was 0.87, while in this study, the Cronbach's *α* was 0.88 and McDonald's *ω* was 0.88.

### 3.3. Data Collection

Data were collected from August 14, 2023, to September 15, 2023, through both face-to-face and online surveys to accommodate the working conditions of shift nurses. For the face-to-face surveys, the researcher visited each department, explained the study, obtained informed consent from participants, distributed questionnaires, and collected completed surveys in sealed envelopes to maintain anonymity. For online surveys, participants who preferred this method were provided with a survey link. To minimize potential survey mode effects, both survey formats employed identical question wording and instructions, and respondent anonymity was guaranteed. In addition, potential response bias was reduced by clearly explaining the study's purpose and necessity and employing a survey instrument with established reliability and validity in the Korean context, which included reverse-worded items to assess the consistency of participants' responses.

### 3.4. Ethical Consideration

This study was approved by the Institutional Review Board (IRB) of Seoul Hanyang University Hospital (IRB no.: 2023-02-042-014). To maintain the anonymity of the research participants, all data except for age were collected using ordinal scales to ensure that specific individuals could not be identified. Participants were informed about the option to participate through online surveys, which further preserved their anonymity. The data from this research will be securely stored on a personal desktop and in a locked filing cabinet for 3 years, after which it will be securely disposed of.

### 3.5. Data Analysis

This study utilized the SPSS/WIN 29.0 (IBM Corp., Armonk, NY, USA) program for data analysis. The specific data analysis methods are as follows:

• General characteristics and the degree of professional autonomy, self-esteem, LMX, TMX, workplace bullying, and organizational silence were analyzed using tallies, percentages, means, and standard deviations. To evaluate the normality of the data, the skewness and kurtosis values were checked, and normality was considered to be satisfied when the absolute value of skewness was less than 2 and the absolute value of kurtosis was less than 7 [46].
• To examine differences in organizational silence according to general characteristics, independent *t*-tests and one-way ANOVA were performed, followed by Scheffé's test for post hoc analysis.
• To assess the potential presence of common method bias, Harman's single-factor test was conducted under the assumption that no single factor accounted for > 50% of the total variance [47].
• To assess the validity of the measurements, exploratory factor analysis (EFA) was conducted. Items with factor loadings below 0.5 were excluded from the analyses.
• Both Cronbach's *α* and McDonald's *ω* [48] were employed to assess the internal consistency of the measurement instrument.
• The associations between professional autonomy, self-esteem, LMX, TMX, workplace bullying, and organizational silence were analyzed using Pearson's correlation coefficient.
• Model 4 test in PROCESS_v4.2 in SPSS (an add-on for SPSS) [49] was used for mediation analysis of workplace bullying. The bias-corrected confidence interval (CI) was set at 95% using the bootstrap method.

## 4. Results

### 4.1. General Characteristics of Participants and Differences in Organizational Silence

Table 1 presents the general characteristics of the participants. Among the 170 nurses, most were female (90.0%), aged 26–29 years (48.8%), unmarried (79.4%), and without religious affiliation (65.3%). The majority were 4-year university graduates (84.1%) and worked in wards (55.9%). Regarding clinical career, the largest group had 5–10 years of experience (32.3%), and for the current work unit tenure, the largest proportion had 1–3 years (35.9%).

Group difference analyses revealed significant variations in organizational silence by clinical career and current work unit experience. Nurses with less than 3 years of clinical career reported significantly higher organizational silence than those with 10 years or more (*F* = 3.18, *p*=0.025), and nurses with less than 1 year of current work unit experience also reported significantly higher than those with 10 years or more (*F* = 4.58, *p*=0.002).

### 4.2. Reliability and Validity

Harman's single-factor test was conducted to check the possibility of common method bias. The results show that the explanatory power of the first factor was 17.9%, which did not exceed 50%, indicating no significant common method bias [47].

EFA was used to evaluate the construct validity of measurements. All measurements were shown to be suitable for EFA, as the Kaiser–Meyer–Olkin (KMO) index was greater than 0.7 and the *p* value of Bartlett's test of sphericity was less than 0.05. A combination of principal component analysis (PCA) and varimax rotation techniques was used to explore the interrelationships between variables. Based on the eigenvalue ≥ 1, items with a factor loading of ≤ 0.50 were excluded and not used in statistical analysis [50].

Cronbach's *α* values for all scales ranged from 0.86 to 0.93, and McDonald's *ω* values ranged from 0.85 to 0.93, indicating satisfactory internal consistency.

### 4.3. Degree of Professional Autonomy, Self-Esteem, LMX, TMX, Workplace Bullying, and Organizational Silence

Table 2 presents the descriptive statistics for professional autonomy, self-esteem, LMX, TMX, workplace bullying, and organizational silence. The mean score for organizational silence was 2.43 ± 0.57. Workplace bullying had a mean of 2.28 ± 0.65. TMX showed the highest mean score (3.75 ± 0.47), followed by LMX (3.22 ± 0.76), self-esteem (3.13 ± 0.53), and professional autonomy (3.07 ± 0.57). Skewness values ranged from −0.42 to 0.38 and kurtosis values ranged from −0.70 to 1.53, all within acceptable thresholds, satisfying the assumption of normality [46].

### 4.4. Correlation Among Variables

Table 3 presents the correlations among the variables. Organizational silence was negatively correlated with professional autonomy (*r* = −0.22, *p*=0.004), self-esteem (*r* = −0.19, *p*=0.013), LMX (*r* = −0.31, *p* < 0.001), and TMX (*r* = −0.36, *p* < 0.001). In addition, a statistically significant positive correlation was found between bullying and organizational silence (*r* = 0.33, *p* < 0.001).

### 4.5. Hypothesis Test Results

**Figure 2:**
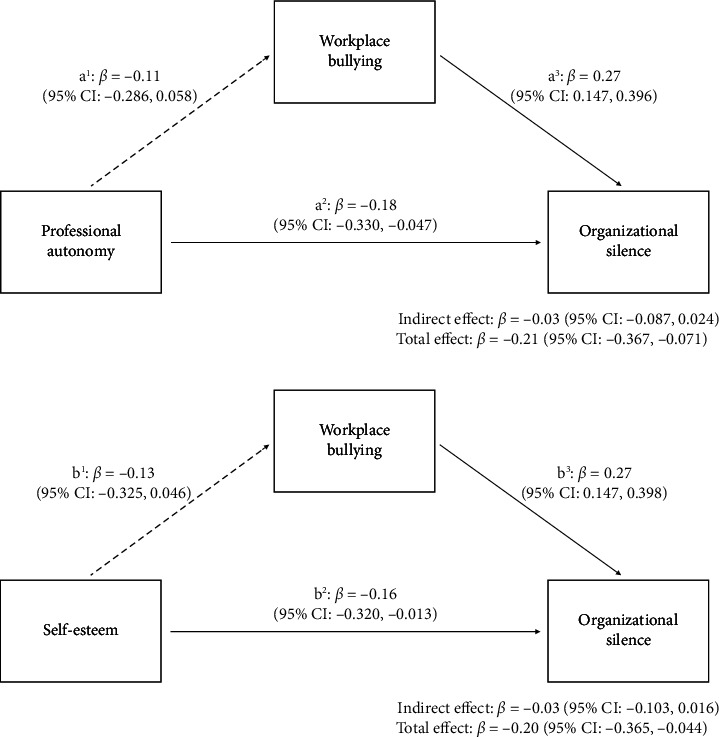
The mediating effect of workplace bullying between personal characteristics and organizational silence.

To test the mediating effect of workplace bullying, PROCESS macro Model 4 was applied (Tables 4 and 5). Professional autonomy (*β* = −0.18, *p*=0.009) and self-esteem (*β* = −0.16, *p*=0.033) directly reduced organizational silence, but their indirect effects through workplace bullying were nonsignificant, leading to the rejection of Hypotheses 1 and 2 (Figure 2).

LMX showed both significant direct (*β* = −0.20, *p* < 0.001) and indirect effects via workplace bullying (*β* = −0.03, 95% CI: [−0.074, −0.001]), supporting Hypothesis 3.

**Figure 3:**
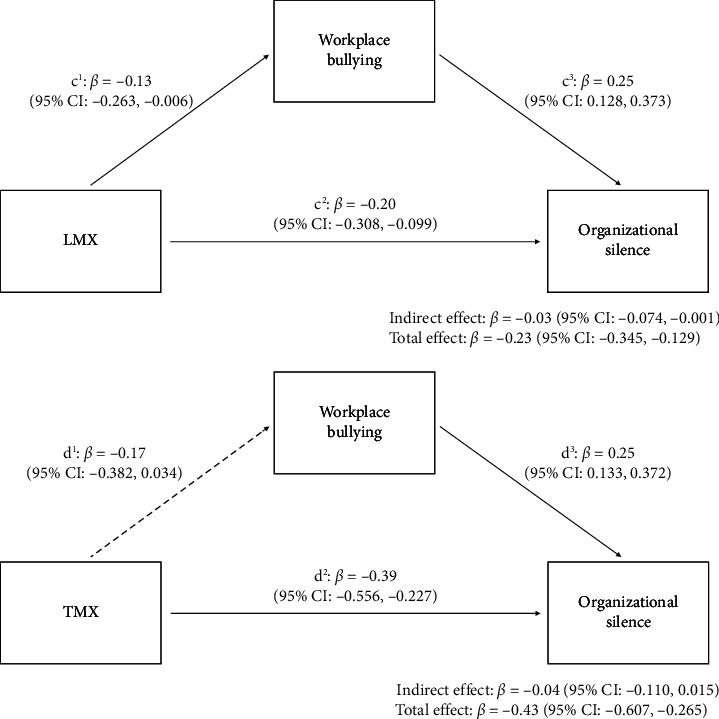
The mediating effect of workplace bullying between organizational situation and organizational silence.

TMX had the strongest direct effect on organizational silence (*β* = −0.39, *p* < 0.001), but no indirect effect, resulting in the rejection of Hypothesis 4 (Figure 3).

## 5. Discussion

According to Milliken et al.'s model [1], the expected negative outcome is a factor that directly affects organizational silence and acts as a mediator between the influencing factor and organizational silence. In this study, only the LMX indirectly influenced organizational silence through workplace bullying, while the other variables did not show such indirect effects.

The role of leadership in organizational silence has been widely recognized. In Korea's vertical and authoritarian organizational culture, relationships with superiors are central to fostering psychological safety, which encourages employee voice and reduces silence [4, 11]. This study found a significant association between LMX and organizational silence. Previous studies have suggested that personalized leadership should be implemented by adjusting leadership styles according to nurses' preferences and needs [51]. LMX is in line with this approach in that it focuses on the quality of the relationship between leaders and each individual member and is expected to be effective in alleviating organizational silence. Asian countries are characterized by high power distance cultures [52], and this hierarchical unit culture acts as a factor that increases the level of organizational silence among nurses [53]. Authoritarian leadership behaviors in such settings may further intensify silence and thus require close attention [54]. Conversely, ethical leadership, marked by fairness and respect, has been shown to reduce both workplace bullying and silence [55, 56]. These findings suggest that positive and qualitative leadership interactions can buffer negative organizational experiences and mitigate silence.

Workplace bullying was significantly associated with organizational silence and served as a potential mediator in the relationship between LMX and silence. These findings are consistent with prior research demonstrating that lower-quality leader–member relationships are associated with increased bullying and that bullying, in turn, contributes to organizational silence by discouraging open communication and trust [57–59]. In hierarchical clinical settings, poor leadership exacerbates this effect by undermining psychological safety and fostering defensive behaviors. Addressing workplace bullying requires both preventive and responsive strategies. The first step in addressing workplace harassment is implementing and communicating an antiharassment policy. This policy should define harassment, outline consequences, establish reporting guidelines, and include regular training to raise awareness [60]. This will help nurses to identify and address bullying more effectively. Workplace bullying management requires both response and preventive strategies.

In this study, professional autonomy and self-esteem, identified as individual-level characteristics, were significantly associated with organizational silence. In addition, the present study also revealed differences in organizational silence according to clinical career and current work unit experience. Consistent with these findings, previous studies have shown that nurses' career experience significantly predicts organizational silence [19, 61]. This suggests that career accumulation can lead to increased understanding of the job and increased autonomy in performing it, and that these changes may also be related to expressive behavior within the organization. In addition, previous studies reporting that nurses' positive psychological capital was associated with their silent behavior support the present findings, suggesting that internal psychological resources such as self-esteem may be related to expressive behavior within an organization [62].

However, workplace bullying did not significantly mediate the relationships between professional autonomy and organizational silence, nor between self-esteem and organizational silence. This suggests that these two personal characteristics function as internal psychological resources that can directly influence self-expressive behavior within the organization, without the need for external negative experiences. Furthermore, workplace bullying is a social phenomenon that is more influenced by the organizational culture and interpersonal dynamics than by individual factors such as autonomy or self-esteem [63]. Given that Korean nursing organizations are characterized by rigid hierarchies and a strong implicit norm of silence [6], it is plausible that organizational culture itself, rather than workplace bullying, exerted a more direct influence on the formation of silence. Accordingly, the mediating role of workplace bullying in linking individual characteristics to silence appears limited.

TMX had the highest mean score and showed the strongest association with organizational silence. This result likely reflects the nature of nursing work in shift-based environments, where continuity of care depends on close collaboration. It also suggests that the quality of interactions among team members plays a meaningful role in reducing nurses' silence. Go [4] also reported that organizational factors were more strongly associated with organizational silence than individual factors. However, the indirect effect of TMX on organizational silence through workplace bullying was not statistically significant. This may be explained by the fact that workplace bullying in Korean nursing organizations predominantly occurs in vertical hierarchical relationships, where senior nurses exercise authority over juniors as part of an entrenched organizational culture [9, 14]. It is expected that TMX, which reflects horizontal relationships and the quality of peer interactions, did not have a significant effect on workplace bullying among Korean nurses, which is shaped by these cultural characteristics. This finding underscores that in strongly hierarchical cultures, peer-level relational quality may play a limited role in mediating the pathway to silence compared to structural organizational dynamics.

Due to the nature of shift work, nurses are highly interdependent with their colleagues. Therefore, the quality of exchanges among team members can directly affect nurses' self-expression behavior. However, in situations where TMX is low, regardless of the presence of workplace bullying, members are highly likely to feel unacknowledged by their colleagues, experience isolation, and remain silent. Furthermore, because workplace bullying in Korean organizational culture typically takes a vertical form within hierarchical relationships [9, 14], the correlation with TMX, which reflects the quality of horizontal relationships, may therefore be theoretically and empirically constrained in this context.

This study is distinguished in that it helps provide a comprehensive understanding of the complex background that leads to organizational silence based on the model of the choice to remain silent framework [1] and specifically identifies the mediating role of workplace bullying in the process by which LMX affects organizational silence. Moreover, the findings underscore the pivotal role of nursing leaders in addressing negative cultural phenomena in nursing organizations, such as silence and workplace bullying, and offer important implications for practical intervention strategies.

LMX has been shown to mitigate both workplace bullying and organizational silencing. Leaders can create a collaborative and innovative work environment by reducing biases and fostering open communication [29]. Leadership development programs, mentorship and peer support initiatives, and work environments that provide both relational and structural support can enhance the leadership competencies of frontline nurse managers [64], and such interventions are crucial for improving negative organizational cultures in nursing. In addition, since bullying mediates the effect of LMX on silence, organizations should establish systematic prevention policies and provide staff education to address bullying incidents. Recently, various approaches have been attempted to prevent workplace bullying. Research has shown that cognitive rehearsal programs, where nurses practice handling bullying situations through role-playing, improve communication and conflict-resolution skills [65]. Furthermore, AI-based nursing education could enhance bullying prevention training, strengthening nurses' ability to address workplace bullying in the workplace more effectively [66]. However, ultimately, policy intervention at the organizational level must take place. Notably, Sweden, Canada, France, and Korea have enacted “Workplace Bullying Prevention Acts,” providing a legal basis for organizational practices [67]. Establishing clear protocols aligned with such international standards could help reduce workplace bullying and organizational silence among nurses.

## 6. Limitations

First, this study was conducted at a tertiary general hospital in Seoul. However, organizational culture among nurses can vary depending on hospital size, nursing staff management, regional characteristics, and hospital type. This can also influence the nature and causes of organizational silence. Therefore, the generalizability of the results of this study is limited, and future research should include comparative and validation efforts across various types and regions of medical institutions. Second, this study focused solely on acquiescent and defensive silence, which are rooted in negative motives, among the reasons for organizational silence. Future studies should include diverse forms of silence, such as prosocial silence, which stems from prosocial motives, to enhance understanding of the factors contributing to organizational silence arising from various motivations. Third, as this study utilized cross-sectional data at a specific point in time, future research employing a longitudinal design is needed to more accurately identify causal relationships. Finally, demographic variables such as age and department type were not considered as potential moderators. Future research should examine whether these variables have moderating effects on the relationships associated with organizational silence.

## 7. Conclusions

The findings suggest that an organizational approach is more effective than an individual-based strategy in alleviating organizational silence. In addition, the findings suggest that strengthening leader–member relationships not only reduces organizational silence but also helps mitigate workplace bullying. Encouraging positive interactions with leaders and fostering open communication empower members to express their views freely and handle conflicts or bullying more effectively.

## Figures and Tables

**Table 1:** General characteristics of participants and differences in organizational silence (*N* = 170).

Characteristics	Categories	*n* (%)	Organizational silence	
			*M* ± SD	*t* or *F* (*p*) Scheffé
Sex	Female	153 (90.0)	2.41 ± 0.56	1.65 (0.100)
	Male	17 (10.0)	2.65 ± 0.61	

Age (years)	≤ 25	30 (17.7)	2.52 ± 0.48	1.35 (0.251)
	26–29	83 (48.8)	2.49 ± 0.61	
	30–39	40 (23.5)	2.31 ± 0.56	
	40–49	13 (7.6)	2.25 ± 0.52	
	≥ 50	4 (2.4)	2.08 ± 0.13	

Marital status	Single	135 (79.4)	2.45 ± 0.58	1.01 (0.313)
	Married	35 (20.6)	2.34 ± 0.53	

Religion	Yes	59 (34.7)	2.45 ± 0.61	0.22 (0.824)
	No	111 (65.3)	2.42 ± 0.55	

Education	College	15 (8.8)	2.35 ± 0.56	0.49 (0.612)
	University	143 (84.1)	2.45 ± 0.57	
	≥ Graduate School	12 (7.1)	2.31 ± 0.56	

Current work unit	Ward	95 (55.9)	2.40 ± 0.53	1.63 (0.184)
	ER	23 (13.5)	2.68 ± 0.67	
	ICU	30 (17.7)	2.38 ± 0.53	
	OR	22 (12.9)	2.40 ± 0.66	

Clinical career (years)	< 3^a^	54 (31.8)	2.56 ± 0.55	3.18 (0.025)^∗^a > d
	3–< 5^b^	35 (20.6)	2.51 ± 0.58	
	5–< 10^c^	55 (32.3)	2.37 ± 0.60	
	≥ 10^d^	26 (15.3)	2.17 ± 0.44	

Current work unit experience (years)	< 1^a^	13 (7.7)	2.76 ± 0.44	4.58 (0.002)^∗^a > e
	1–< 3^b^	61 (35.9)	2.52 ± 0.55	
	3–< 5^c^	39 (22.9)	2.50 ± 0.60	
	5–< 10^d^	48 (28.2)	2.27 ± 0.52	
	≥ 10^e^	9 (5.3)	1.92 ± 0.50	

*Note: M* = mean.

Abbreviations: ER = emergency room; ICU = intensive care unit; OR = operating room; SD = standard deviation.

^∗^Scheffe' test.

^a–e^Different superscript letters indicate significant differences between groups based on post hoc analysis (*p* < 0.05).

**Table 2:** The descriptive statistics of the study variables (*N* = 170).

Variable	Range	Min	Max	*M* ± SD	Skewness	Kurtosis
Professional autonomy	1–5	1.36	5.00	3.07 ± 0.57	0.36	1.53
Self-esteem	1–4	1.88	4.00	3.13 ± 0.53	−0.20	−0.70
LMX	1–5	1.00	5.00	3.22 ± 0.76	−0.42	0.32
TMX	1–5	2.40	5.00	3.75 ± 0.47	0.00	0.07
Workplace bullying	1–5	1.00	3.80	2.28 ± 0.65	0.14	−0.63
Organizational silence	1–5	1.00	4.11	2.43 ± 0.57	0.38	0.38

*Note: M* = mean.

Abbreviations: LMX = leader–member exchange; SD = standard deviation; TMX = team-member exchange.

**Table 3:** Correlation among the study variables (*N* = 170).

Variable	1	2	3	4	5	6
	*r* (*p*)	*r* (*p*)	*r* (*p*)	*r* (*p*)	*r* (*p*)	*r* (*p*)
1. Professional autonomy						
2. Self-esteem	0.20 (0.007)					
3. LMX	0.35 (< 0.001)	0.06 (0.407)				
4. TMX	0.26 (< 0.001)	0.28 (< 0.001)	0.23 (0.002)			
5. Workplace bullying	−0.10 (0.195)	−0.11 (0.139)	−0.15 (0.040)	−0.12 (0.100)		
6. Organizational silence	−22 (0.004)	−0.19 (0.013)	−0.31 (< 0.001)	−0.36 (< 0.001)	0.33 (< 0.001)	

Abbreviations: LMX = leader–member exchange; TMX = team-member exchange.

**Table 4:** The mediating effect of workplace bullying between personal characteristics and organizational silence.

**Path**		**Effect**	***β***	**SE**	***t* (** **p** **)**	**LLCI**	**ULCI**

PA ⟶ WB	a^1^	Direct	−0.11	0.08	−1.30 (0.194)	−0.286	0.058
PA ⟶ OS	a^2^		−0.18	0.07	−2.63 (0.009)	−0.330	−0.047
WB ⟶ OS	a^3^		0.27	0.06	4.32 (< 0.001)	0.147	0.396

PA ⟶ WB ⟶ OS		Indirect	−0.03	0.02^∗^		−0.087^∗^	0.024^∗^
		Total	−0.21	0.07^∗^	−2.93 (0.003)	−0.367^∗^	−0.071^∗^

SE ⟶ WB	b^1^	Direct	−0.13	0.09	−1.48 (0.139)	−0.325	0.046
SE ⟶ OS	b^2^		−0.16	0.07	−2.14 (0.033)	−0.320	−0.013
WB ⟶ OS	b^3^		0.27	0.06	4.30 (< 0.001)	0.147	0.398

SE ⟶ WB ⟶ OS		Indirect	−0.03	0.02^∗^		−0.103^∗^	0.016^∗^
		Total	−0.20	0.08^∗^	−2.52 (0.012)	−0.365^∗^	−0.044^∗^

Abbreviations: LLCI = lower limit confidence interval; OS = organizational silence; PA = professional autonomy; SE = self-esteem; ULCI = upper limit confidence interval; WB = workplace bullying.

^∗^Bootstrapping method.

**Table 5:** The mediating effect of workplace bullying between organizational situation and organizational silence.

**Path**		**Effect**	***β***	**SE**	***t* (** **p** **)**	**LLCI**	**ULCI**

LMX ⟶ WB	c^1^	Direct	−0.13	0.06	−2.07 (0.039)	−0.263	−0.006
LMX ⟶ OS	c^2^		−0.20	0.05	−3.84 (< 0.001)	−0.308	−0.099
WB ⟶ OS	c^3^		0.25	0.06	4.04 (< 0.001)	0.128	0.373

LMX ⟶ WB ⟶ OS		Indirect	−0.03	0.01^∗^		−0.074^∗^	−0.001^∗^
		Total	−0.23	0.05^∗^	−4.34 (< 0.001)	−0.345^∗^	−0.129^∗^

TMX ⟶ WB	d^1^	Direct	−0.17	0.10	−1.65 (0.100)	−0.382	0.034
TMX ⟶ OS	d^2^		−0.39	0.08	−4.70 (< 0.001)	−0.556	−0.227
WB ⟶ OS	d^3^		0.25	0.06	4.17 (< 0.001)	0.133	0.372

TMX ⟶ WB ⟶ OS		Indirect	−0.04	0.03^∗^		−0.110^∗^	0.015^∗^
		Total	−0.43	0.08^∗^	−5.03 (< 0.001)	−0.607^∗^	−0.265^∗^

Abbreviations: LLCI = lower limit confidence interval; LMX = leader–member exchange; OS = organizational silence; TMX = team-member exchange; ULCI = upper limit confidence interval; WB = workplace bullying.

^∗^Bootstrapping method.

## Data Availability

Data are available for the appropriate reason by requesting to the corresponding author.
